# Calcinosis Prevalence in Autoimmune Connective Tissue Diseases—A Retrospective Study

**DOI:** 10.3390/jcm13123428

**Published:** 2024-06-12

**Authors:** Lili Róbert, Krisztián Németh, Márta Marschalkó, Péter Holló, Bernadett Hidvégi

**Affiliations:** Department of Dermatology, Venereology and Dermatooncology, Semmelweis University, H-1085 Budapest, Hungary

**Keywords:** calcinosis cutis, prevalence, epidemiology, autoimmune connective tissue diseases, systemic sclerosis, dermatomyositis

## Abstract

**Background/Objectives**: Calcinosis cutis is the deposition of insoluble calcium salts, which may cause inflammation, ulceration, pain, and restricted joint mobility. It rarely develops in damaged tissues (dystrophic subtype), most frequently in autoimmune connective tissue diseases (CTDs), but there is very limited data on the prevalence. Also, therapy remains an unsolved issue. In this study, we aimed to collect data on the prevalence of calcinosis in CTD patients to highlight that it is a considerable problem. **Methods**: A retrospective study was conducted in our department to assess the epidemiology of dystrophic calcinosis in CTDs between January 2003 and January 2024. **Results**: A total of 839 CTD patients were identified, of whom 56 had calcinosis (6.67%). The mean age of the calcinosis patients at diagnosis of underlying CTD was 41.16 ± 19.47 years. The mean time interval from the onset of calcinosis was 5.96 ± 8.62 years. Systemic sclerosis was the most common CTD complicated by calcinosis (n = 22). **Conclusions**: Our results are comparable to those reported previously in the literature. Although calcinosis is rare in the overall population, it is a present and unsolved problem in CTD patients. Therefore, further studies are needed on the factors involved in the development and progression of calcinosis as well as its treatment.

## 1. Introduction

Calcinosis cutis is the deposition of insoluble calcium salts surrounded by inflammatory cells in the soft tissues of the skin, which may occur as a rare complication of various entities [1,2]. It can be subdivided into four subtypes based on the mechanism of formation (dystrophic, metastatic, idiopathic, and iatrogenic). The most common dystrophic subtype develops in damaged or devitalized tissues in the absence of calcium and phosphate metabolism disturbances [3]. Autoimmune connective tissue diseases (CTD) are the most numerous group in which dystrophic calcinosis may be present. It has been described in dermatomyositis (DM), juvenile dermatomyositis (JDM), polymyositis, systemic sclerosis (SSc), systemic lupus erythematosus (SLE), mixed connective tissue disease (MCTD), as well as Sjögren’s syndrome and rheumatoid arthritis, but in theory, it may develop in any CTD. The CTDs most commonly associated with calcinosis are DM and SSc [4]. Pathogenesis is not known in detail [5]. There are several possible mechanisms contributing to the formation of insoluble crystal deposits. These include cellular membrane damage leading to elevated calcium and phosphate concentrations in the mitochondria [4], acidic pH causing the suppression of calcification inhibitor molecules [1], phosphate bound to denatured proteins [6,7], chronic inflammation [8,9,10,11], recurrent microtrauma [12,13,14], tissue hypoxia [15,16,17,18,19,20,21,22,23], and disturbed extracellular matrix mineralization metabolism [2,24,25,26,27,28,29,30,31]. It is hypothesized that in SSc, ischemia is the main pathogenetic factor, while in DM, inflammation leads to calcium salt precipitation [32].

Calcinosis presents clinically as firm, whitish or skin-colored papules, nodules, or subcutaneous masses. Complications include pain, ulceration, infection, excretion of chalky material, restricted mobility of the adjacent joints, muscle atrophy, and disfigurement. Therefore, calcinosis may significantly affect the quality of life and present as the leading complaint in a subset of CTD patients. It can be diagnosed by physical examination. In cases of doubt, plain radiography is the first-line diagnostic method, considering its accessibility and short time requirement [33]. Other diagnostic and confirmatory possibilities include ultrasonography [34], multidetector computed tomography, dual-energy computed tomography, bone scintigraphy, and magnetic resonance imaging [35,36,37,38]. There is very limited data in the literature on the prevalence of calcinosis cutis, and treatment remains an unsolved issue. Several pharmacological and procedural therapies have been published. However, there are no evidence-based guidelines to aid clinicians in their management, and surgical excision remains the mainstay of therapy [39]. Therapeutic recommendations are based on smaller calcinosis populations, mainly complicating SSc and DM, and none of the therapeutic methods have shown universal efficacy. Treatment should be tailored to each patient based on extent, localization, inflammation, and comorbidities. The variability of individual responses to therapy is high. Traineau et al. [40] summarized the current experience in a review paper. The recommended therapies are shown in Table 1. Other drugs with even less experience or less favorable results are probenecid [5,41,42], aluminum hydroxide [5,43], ceftriaxone [44], warfarin [45], thalidomide [46], intravenous or oral sodium thiosulfate [47], and intralesional corticosteroids [48,49].

Given that calcinosis is a current and unsolved problem in terms of its management, there is also little data on its prevalence. In this retrospective study, our aim was to provide data on the epidemiology of calcinosis complicating CTDs and to draw attention to it. 

## 2. Materials and Methods

In this retrospective observational study, our objective was to assess the prevalence of calcinosis cutis in CTD patients at our Department of Dermatology between 2003 and 2024. We reviewed our system of medical reporting and data retrieval (eMedSolution (T-Systems Hungary Ltd., Budapest, Hungary, Version: 2024/Q1/1)) for cases coded as CTDs according to the International Classification of Diseases between 1 January 2003 and 1 January 2024. The CTDs investigated included SSc (diffuse cutaneous systemic sclerosis (dcSSc) and limited cutaneous systemic sclerosis (lcSSc)), DM, JDM, SLE, MCTD, undifferentiated connective tissue disease (UCTD), and overlap connective tissue syndromes. The initial search identified 1576 patients coded as having a CTD. That was followed by a manual assessment to determine whether the diagnosis of the CTD was confirmed. Cases where the diagnosis of the CTD was incorrect or not confirmed by an expert clinician (an expert dermatologist, immunologist, or rheumatologist) were excluded. Duplicate cases classified as multiple diagnoses were counted once. The presence of calcinosis was manually evaluated. We also searched for cases in the same database coded as calcinosis cutis. That followed a manual search for confirmed calcinosis (by an expert, radiological imaging, or histopathology) complicating a CTD. Detailed data collection was performed on this merged population. The clinical, radiological, and immune serology records were evaluated. The following data were extracted: sex, underlying CTD, age at diagnosis of CTD, age at diagnosis of calcinosis, time interval between the diagnosis of the CTD and onset of calcinosis, location of calcinosis, ulceration at the calcinosis site, detectable autoantibodies in the blood sera of calcinosis patients, histopathological reports, and confirmatory imaging technique reports (plain radiography, ultrasonography, magnetic resonance imaging, and computed tomography).

## 3. Results

**Figure 1:**
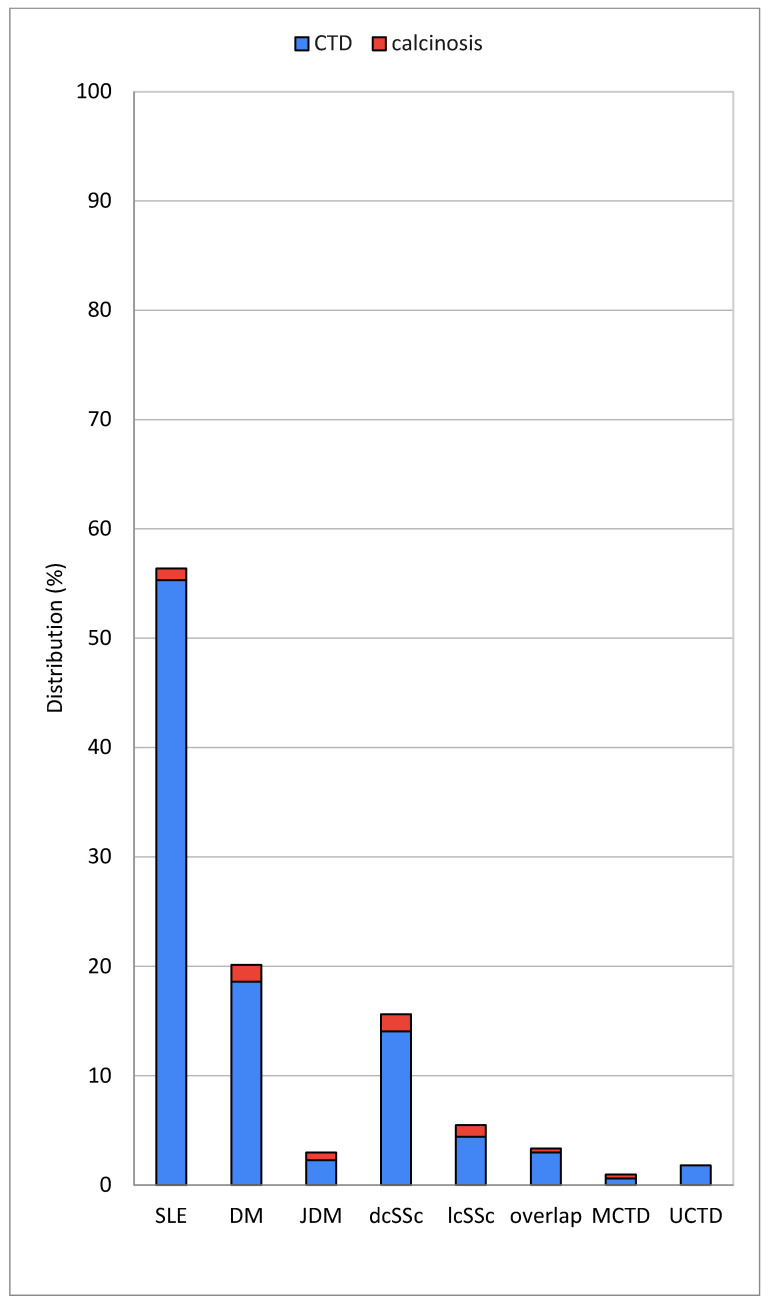
Distribution of different CTDs and associated calcinosis in our study population.

A total of 839 patients had verified CTD (male/female ratio = 680:159), among which SLE was the most common (n = 464). Calcinosis was detected in 56/839 patients (male/female ratio = 11:45), representing an overall prevalence of 6.67% in our CTD population. The mean age of the calcinosis patients at the diagnosis of the underlying CTD was 41.16 ± 19.47 years (range 4–75). The mean age at onset of calcinosis was 47.49 ± 20.91 years (range 4–75). The mean time interval between the diagnosis of the CTD and the onset of calcinosis was 5.96 ± 8.62 years (range −11–33). SSc was the most common CTD complicated by calcinosis (22/155 patients). The distribution of different CTDs and associated calcinosis in our study population is shown in Figure 1.

### 3.1. Location

Upper extremities were most commonly affected by calcinosis in 30/56 cases, lower extremities (including the buttocks) were affected in 30/56 cases, 10/56 patients had calcinosis on the trunk, 14/56 patients had calcinosis on more than one part of the body, and 22/56 patients had ulceration at the site of calcinosis (39.28%).

### 3.2. Diagnosis of Calcinosis

In all cases, calcinosis was diagnosed by an expert physician (an expert dermatologist, immunologist, or rheumatologist). Plain radiography, ultrasonography, histopathology, and magnetic resonance imaging confirmed the diagnosis in 22/56, 8/56, 9/56, and 3/56 cases, respectively. In 9/56 cases, calcium deposition was confirmed by two different modalities.

### 3.3. Autoantibodies

Immunserology testing was carried out in 55/56 calcinosis patients. Anti-nuclear antibodies were most frequently detected in 50/56 patients (89.28%), and 43/56 (76.78%) of patients had a titer positivity of at least 1:160. Other common autoantibodies detected were anti-chromatin, extractable nuclear antigen, and anti-Sjögren’s syndrome-related antigen (SSA), in 42.85%, 33.92%, and 25%, respectively. Anti-cytoplasm positivity was detected on Hep2 cells with the immunofluorescence technique in 17.85% of the calcinosis patients. 

The distribution of the autoantibodies was as follows. In the scleroderma group (dcSSc and lcSSc) complicated with calcinosis, anti-Pm/Scl, anti-centromere, and anti-Scl-70 were positive in 7/22, 6/22, and 4/22 patients, respectively. In the dermatomyositis group (DM and JDM), 3/19 patients had anti-cytoplasm, while anti-Mi2-α, anti-Pl-12, anti-Pl-7, anti-NXP2, and anti-Tif1γ was detected in 3/19, 2/19, 1/19, 1/19, and 1/19 patients, respectively. In SLE patients with calcinosis, anti-nucleosome was positive in 6/9 patients, anti-dsDNA and SSA in 5/9 patients, anti-RNP/Sm in 4/9 patients, and anti-β2GPI in 3/9 patients. No correlation was found between the presence of autoantibodies and calcinosis. 

### 3.4. Characteristics of the CTDs

#### 3.4.1. Systemic Sclerosis

A total of 155 patients had SSc (male/female ratio = 28:127). DcSSc and lcSSc were diagnosed in 118/155 and 37/155 patients, respectively. The results showed 22/155 patients (14.19%) had calcinosis, of whom 13/22 patients had dcSSc and 9/22 had lcSSc. This represents an 11.02% calcinosis frequency in dcSSc and 24.32% in lcSSc. In dcSSc, lower extremities were most commonly involved by calcification in 8/13 patients, and upper extremities and trunk were affected in 5/13 and 1/13 cases, respectively. In 2/13 patients, more than one area was involved. Ulceration of the calcified lesions was observed in 7/13 patients. In lcSSc, upper extremities were most commonly involved with calcinosis in 6/9 patients, and lower extremities were affected in 4/9 cases. One patient had more than one calcinosis site. Ulceration was detected in 4/9 cases.

#### 3.4.2. Dermatomyositis

A total of 175 patients had adult or juvenile DM (male/female ratio = 58:117). DM was diagnosed in 156/175 patients and JDM in 19/175 patients. The results showed 19/175 patients had calcinosis, and it complicated DM in 13/19 and JDM in 6/19 cases. This represents an 8.33% calcinosis frequency in DM and 31.58% in JDM. In DM complicated with calcinosis, the upper extremities were most commonly involved by calcinosis in 8/13 patients, and the trunk and lower extremities were affected in 6/13 and 4/13 cases, respectively. In addition, 4/13 patients had more than one affected area. Ulcerated calcinosis was seen in only one patient. In JDM, upper extremities were most commonly involved by calcinosis in 4/6 patients, and lower extremities and trunk were affected in 2/6 and 1/6 cases, respectively. One patient had more than one affected area, and one patient had ulceration at the calcinosis site.

#### 3.4.3. Systemic Lupus Erythematosus

A total of 464 patients had SLE (male/female ratio = 44:420). The results showed 9/464 patients (1.94%) had calcinosis. Upper and lower extremities were equally frequently involved in 6/9 patients, and the trunk was affected in 1/9 patients. In addition, 3/9 patients had more than one affected area. Ulcerated calcinosis was detected in 6/9 patients.

#### 3.4.4. Overlap Connective Tissue Syndromes

A total of 25 patients had an overlap connective tissue syndrome (male/female ratio = 3:22). The results showed 3/25 patients (12%) had calcinosis (all females). The diagnosis of the patients was SLE/RA, DM/SLE, and DM/SSc overlap. Lower extremities were involved in all patients, upper extremities in 2/3 patients, and trunk in 1/3 patients; 2/3 patients had more than one area involved. Two patients had ulcerations at the calcinosis site.

#### 3.4.5. Mixed Connective Tissue Disease

Five patients had MCTD, and they were all females. The results showed 3/5 patients had calcinosis (60%). Lower extremities were involved in 2/3 patients, and upper extremities in 1/3 patients. One patient had ulceration at the calcinosis site.

#### 3.4.6. Undifferentiated Connective Tissue Disease

A total of 15 patients had UCTD (male/female ratio = 2:13). None of them had calcinosis. The characteristics of our calcinosis population are presented in Table 2.

## 4. Discussion

In our study, the following observations were made. Calcinosis cutis is a considerable problem in CTD patients in terms of its prevalence. We found 6.67% frequency in our CTD population. SSc was most commonly complicated by calcinosis. The LcSSc subtype was much rarer in the CTD population compared to the dcSSc subtype. The majority of the SSc patients with calcinosis were female. Ulceration was relatively frequent in both the lcSSc and dcSSc subtypes (44.44 and 53.85%). Calcification was seen more frequently in the lcSSc subtype and showed an earlier onset. Lower extremities were mostly affected in dcSSc, while the upper extremities were affected in the lcSSc subtype. Although DM was far more common in females, calcinosis in DM affected both sexes almost equally. In JDM, calcification was detected in about one-third of the patients, almost only males. Calcinosis showed an early onset in the DM and JDM populations, mainly on the upper extremities, but ulceration occurred quite rarely. We hypothesize that this rare ulcerative tendency may be due to the deeper localization of calcinosis in the dermatomyositis group. SLE was relatively rarely complicated by calcinosis; in these cases, it showed a late onset, and ulceration was rather common (66.67%). Both extremities were equally frequently affected. In overlap syndromes, calcinosis showed a late onset on the lower extremities with ulceration. We had a few MCTD patients, and calcinosis developed in most of them with an early onset. We detected no calcinosis in UCTD patients. We did not observe any significant correlation between laboratory or clinical findings in the calcinosis patients compared to those patients with CTDs without calcinosis. However, we would like to highlight that silent microcalcinosis was not assessed, and the retrospective nature of the study did not allow further data collection in those aspects where no clinical data was available in the medical reports.

In contextualization of our results with those in the literature, calcinosis was found in 18 to 49% of SSc patients in previously reported papers [3,29,64,65,66,67]. We found a slightly lower prevalence of 14.19%. In a study of 7056 SSc patients, the authors reported a 22% frequency (17% in dcSSc and 25% in lcSSc). We found a similar frequency of 11.02% in dcSSc and 24.32% in lcSSc. A study also reported a high frequency (50%) of silent calcinosis in early scleroderma [68]. In previously published papers, calcinosis was found to most commonly develop more than 10 years after the diagnosis of SSc [5,69], although it may precede the diagnosis in lcSSc [50,65]. In our population, an earlier onset was detected after a mean time interval of 6.31 years in dcSSc and 2.25 years in lcSSc. Lesions have been reported to often be small but may involve large areas [70]. The most common locations reported have been the sites of recurrent microtrauma [4,50,71], such as the hand in 65 to 83%, particularly the thumbs (19%) [72], followed by proximal upper extremities, proximal lower extremities, and hip in 27%, 27%, and 6.7%, respectively [5,33,50,65,73]. Calcinosis is complicated by swelling and functional impairment [1,74,75], and it appears to be a significant factor in morbidity [39]. Associations with older age [3,68], longer disease duration, lcSSc subtype, pitting scars [20,22,76], acroosteolysis, telangiectasia, cardiac involvement, gastrointestinal involvement, pulmonary involvement, arthritis, anti-centromere antibodies, anti-PM/Scl antibodies, and anti-cardiolipin autoantibodies have been found. They found the strongest association with digital ulcers and osteoporosis [2,29,39,64,65,66,67,77,78]. Other authors have reported associations with the dcSSc subtype, anti-scl-70, and ANA positivity [65,79]. 

In DM, calcinosis may present as small papules or nodules in the fascia or in the intramuscular connective tissue [80] or even cause an exoskeleton [1,81]. Extremities and the trunk are mostly affected [4,50,82]. It is reported to be the most frequent in JDM (44 to 70%) and shows an earlier onset after 2 to 3 years from the diagnosis of JDM [4,50,83] compared to adult DM and PM, where a calcinosis frequency of 20 to 37% and 3.3% was found, respectively, after a mean duration of 8 years after primary diagnosis [4,50,83,84,85,86]. In comparison, we found an 8.33% prevalence in DM and 31.58% in JDM after a shorter mean period from primary diagnosis of 2.85 and 2.17 years, respectively. Early aggressive therapy seems to reduce the calcinosis burden in JDM [83,87]. Calcinosis was unrelated to disease flare [88] but more frequent in uncontrolled disease, delayed diagnosis [89], longer disease duration [90], dysphagia [91], and Gottron’s sign [84]. Associated autoantibodies were anti-NXP2/MJ and PM/Scl [84,90]. Calcinosis in DM may be a paraneoplastic sign, especially in hematologic diseases [92], and extremely rare malignant tumors may originate from calcinosis lesions [93,94].

Papers have reported a rare presence of calcinosis in SLE [6,50,95], commonly an incidental finding on imaging techniques [6]. In a retrospective study by Tiao et al., 10/148 patients had calcinosis (6.76%). All were female, and calcinosis was not associated with a disease flare. The mean age was 53 ± 17.4 years [96]. Another paper reported the late onset of calcinosis after a mean time interval of 21.5 years after the diagnosis of SLE [6,50]. We found a lower prevalence (1.94%) in a roughly four-times bigger SLE population (9/464). Calcinosis developed comparably late, after a mean time interval of 15.22 years after the diagnosis of SLE. The most common location was reported to be the lower extremities [4,96,97]. Other papers have described interphalangeal joints, forearms, elbows, buttocks, peri-auricular, and subcutaneous areas as potential locations [98,99,100]. In the study by Tiao et al., the Raynaud phenomenon was frequent, and they found a higher titer of ANA autoantibody (minimum 1:160). Frequently associated autoantibodies were anti-RNP, anti-Smith, anti-Ro, and anti-dsDNA in 7/10, 6/10, 7/10, and 7/10, respectively [96].

Considering overlap syndromes, calcinosis is uncommon and located on the extremities, hands, and feet [50,101]. UCTD is also rarely complicated by calcinosis formation after a mean time interval of 2.7 years from the primary diagnosis, mainly on the extremities, hands, and feet [102,103]. MCTD has also been found to be uncommonly associated with calcinosis 6 years after the diagnosis of MCTD, affecting the extremities or buttocks [50,104]. Our population of overlap syndromes, UCTD, and MCTD patients was rather small to conclude any statistically significant statement. Potentially, insoluble calcium salts may be precipitated in all CTDs. Calcinotic lesions may become inflamed, and ulceration has been described in half of the cases [73].

### Limitations

The limitation of our study is its retrospective nature, which did not allow standardized data processing. We could only extract data from the clinical reports of the attending physicians and imaging results. However, ours is one of the few comprehensive studies presenting a large CTD patient group and a considerable calcinosis population.

## 5. Conclusions

Calcinosis cutis is a long-known but scarcely investigated disorder, although it affects many CTD patients. It is a rare condition in the overall population, but papers have reported about a frequency of 18 to 49% in SSc patients, which cannot be regarded as rare in this specific population [3,29,64,65,66,67]. Proper estimation of the prevalence is difficult due to different reasons. Prospective studies would be desirable, but considering the relative rarity of this condition and its wide background heterogeneity, studies are difficult to conduct. On the other hand, calcinosis may remain silent, and in such cases, it is not routinely screened, therefore remaining undiscovered. Calcinosis is mainly examined when it causes complaints, e.g., palpable nodules, pain, ulceration, discharge, joint movement restriction, or disfigurement. In cases of silent, undiscovered calcinosis, it would be unreasonable to screen the whole body for microcalcinoses and reassess the screening as the disease progresses, or even on a regular basis, considering the radiation exposure to patients. It is also doubtful if silent microcalcinosis has any relevance to management. It is often an accidental finding on plain radiography performed for other reasons, causing no complaints from the patients. On the other hand, silent calcinosis may serve as a nidus for further deposit enlargement, resulting in pain or ulceration of the overlying skin or restricted function of the adjacent joints. Considering the accidentally discovered microcalcinosis cases, we hypothesize that the actual prevalence may be much more frequent than reported. It has not yet been decided whether or not calcinosis is a sign of a more severe disease, but papers have not found any association with disease flare. Potentially, it could be a marker of a not-well-controlled disease, ischemia, or inflammation, as impaired blood supply and inflammation have been reported in the literature to be risk factors [32]. Given the potential later complications caused by calcified deposits, we would consider it desirable to treat even silent calcinosis to prevent further damage to the surrounding tissues.

With this paper, we hope to contribute to the body of knowledge relating to CTDs, mainly with respect to calcinosis, drawing attention to a problem that remains unsolved so far. To the best of our knowledge, there is very limited data published on the epidemiology of calcinosis in CTDs. Our results are comparable to the data reported previously. In this retrospective study, we found an overall prevalence of 6.67% calcinosis in CTD patients, which shows a considerable complication in this group of patients. This number shows cases where calcinosis was rather bothersome or visible, which led to the recognition of calcinosis. The lower frequency of calcinosis than that published previously is due to the retrospective nature of the study, which did not allow further radiological investigation for calcinosis, with only clinical reports of the attending physician being available. Also, many of the patients were lost on follow-up a couple of months or years after the primary diagnosis, making it impossible to detect a later onset of calcinosis, although it is usually formed years after the primary diagnosis of CTD. We found SSc to be most frequently complicated by calcinosis, especially the lcSSc subtype. DM was the only group where both sexes were equally affected by calcinosis, with other CTDs showing a marked female predominance. Ulceration was relatively frequent in SLE and SSc but rare in DM. We hypothesize that it could be caused by the deeper localization of calcinosis in DM and ischemia in SSc, which could lead to an ulcerative tendency. As it seems to be related to ischemia, the detection of calcinosis may show impaired blood supply and warrant therapeutic intervention targeting perfusion improvement. Further studies on this topic are needed.

## Figures and Tables

**Table 1:** Therapeutic options in the treatment of calcinosis in CTDs.

Therapy	Mechanism of Action	Level of Evidence	Major Side Effects
Diltiazem [5,50]	Reduced intracellular calcium influx via calcium channel blockage	IV	Peripheral edema, headache, dizziness, low blood pressure, arrhythmia, gastrointestinal
Bisphosphonates (pamidronate, alendronate, risedronate, and etidronate) [5,51,52,53]	Inhibition of osteoclast activity and a decrease in serum calcium level	IV	Gastrointestinal, musculoskeletal, mandibular necrosis, dizziness, headache
Intralesional or topical sodium thiosulfate [47]	Antioxidant, chelator, and vasodilator	IV	Gastrointestinal, headache, blood chemistry abnormalities, high or low blood pressure
Intravenous immunoglobulin [54,55,56]	Inhibition of inflammation	IV	Infections, thromboembolism, anaphylaxis, headache, weakness, gastrointestinal
Colchicine [50,57]	Inhibition of inflammation (decreased chemotaxis of white blood cells)	IV	Anemia, cytopenia, gastrointestinal, rhabdomyolysis
Minocycline [5,58]	Inhibition of inflammation via matrix metalloproteinase inhibition and binding of calcium	IV	Photosensitivity, hepatotoxicity, bone growth suppression in children, and idiopathic intracranial hypertension
Rituximab [59,60]	Inhibition of inflammation via inhibition of CD20 surface antigen, which may play a role in calcium influx	IV	Anaphylaxis, infections
Surgical excision [5,50]	Removal of calcified deposits	IV	Wound infection
Carbon dioxide laser [61]	Destruction of deposits	IV	Post-inflammatory hyperpigmentation, scarring
Extracorporeal shock wave lithotripsy [62,63]	Destruction of deposits	IV	None

**Table 2:** Characteristics of our calcinosis population.

CTD	Calcinosis (n)	Male (n)	Female (n)	Mean Age at Diagnosis (y) (±SD)	Mean Age at Calcinosis Onset (y) (±SD)	Time Interval to Calcinosis (y) (±SD)
DM	13	4	9	52.23 (±14.70) (range 21–74)	55.08 (±15.63) (range 21–74)	2.85 (±4.69) (range 0–17)
JDM	6	5	1	7 (±2.37) (range 4–11)	9.17 (±3.31) (range 4–14)	2.17 (±1.17) (range 0–3)
dcSSc	13	1	12	39.85 (±16.32) (range 10–69)	45.85 (±17.45) (range 11–71)	6.31 (±6.75) (range −7–18)
lcSSc	9	0	9	53.89 (±17.13) (range 17–75)	60.75 (±21.82) (range 50–75)	2.25 (±4) (range 0–12)
SLE	9	1	8	35 (±15.84) (range 16–65)	50.22 (±16.38) (range 27–70)	15.22 (±11.97) (range 0–33)
Overlap	3	0	3	45 (±4) (range 41–49)	55.67 (±15.57) (range 41–72)	10.67 (±14.36) (range 0–27)
MCTD	3	0	3	43.67 (±9.02) (range 35–53)	46.67 (±19.65) range (24–59)	3 (±13.53) (range −11–65)
UCTD	0	0	0	-	-	-

Abbreviations: CTD: autoimmune connective tissue disease, dcSSc: diffuse cutaneous systemic sclerosis, DM: dermatomyositis, JDM: juvenile dermatomyositis, lcSSc: limited cutaneous systemic sclerosis, MCTD: mixed connective tissue disease, n: number of patients, SD: standard deviation, SLE: systemic lupus erythematosus, UCTD: undifferentiated connective tissue disease, y: years.

## Data Availability

The data that support the findings of this study are available on request from the corresponding author. The data are not publicly available due to privacy or ethical restrictions.
